# Effects of Gambisan in overweight adults and adults with obesity: A retrospective chart review

**DOI:** 10.1097/MD.0000000000018060

**Published:** 2019-11-22

**Authors:** Dae-Hyun Jo, Seunghoon Lee, Jae-Dong Lee

**Affiliations:** a Department of Acupuncture and Moxibustion, College of Korean Medicine, Kyung Hee University; b Department of Korean Medicine, Geumwang Health Subcenter, Bureau of Health Policy, Ministry of Health and Welfare, Seoul, Republic of Korea.

**Keywords:** retrospective chart review, Traditional Korean Medicine, herbal medicine, Gambisan, weight loss, obesity, overweight

## Abstract

**Objective:**

A retrospective chart review was conducted to explore the effect of Gambisan, a granular extract of novel herbal medicine, for short-term (≤16 weeks) weight loss in adults who are overweight and those with obesity.

**Methods:**

Outpatients of Kyung Hee University Korean Medicine Hospital (Seoul, Korea) who took Gambisan and underwent bioelectric impedance analysis were selected (Jan 2011 to Dec 2015); their electronic medical records and clinical charts were retrospectively reviewed. The effectiveness of Gambisan was primarily evaluated by comparing body weight (BW) at baseline and endpoint, using paired *t* tests; the safety of Gambisan was evaluated on the basis of adverse events (AEs) experienced by patients.

**Results:**

Two hundred five patients were included in this study. The study population exhibited a significant reduction in BW (73.69 ± 14.49 kg to 69.01 ± 13.20 kg, *P* < .001) as well as percentage body fat (37.38 ± 5.38% to 34.50 ± 5.83%, *P* < .001). Moreover, 111 (54.1%) patients achieved modest weight loss (≥5%), while 35 (17.1%) achieved ≥10% weight loss. Furthermore, Gambisan induced significant reduction of BW in all subgroups (body mass index, sex, prescribed duration, and dosage). Among 139 patients with available data, 79 (56.8%) reported loss-of-appetite. In addition, 120 (mostly mild) AEs were reported in 69 (49.6%) patients, and the most frequent AEs were nausea, palpitation, and insomnia.

**Discussion:**

Despite limitations in interpreting the results of this retrospective medical record review, Gambisan induced statistically and clinically meaningful weight loss with a tolerable level of AEs. Based on the findings of this review, further well-designed clinical trials are warranted.

## 1 Introduction

It has been estimated that 1.9 billion people globally are overweight, of which 650 million exhibit obesity.^[1]^ In South Korea, the rate of obesity in the population has been constantly increasing since 1998 and is now estimated to be 37.9% in men and 25.9% in women.^[2]^ Because obesity induces a battery of complications, from hypertension and type 2 diabetes to deadly stroke and coronary syndromes,^[3]^ management of obesity has attracted significant attention. However, correcting dietary and exercise habits requires significant effort and often results in disappointing reward after behavioral modification^[4]^; thus, pharmaceutical intervention has been highlighted as a complementary option. Although orlistat, sibutramine, phentermine, and rimonabant have been prescribed for individuals with obesity, they have a limited weight-maintaining effect^[5]^ or an unfavorable risk-benefit ratio, with various adverse events (AEs) or harmful impact.^[6–8]^ This has prompted demands for novel agents that are based on components from natural plants or herbal decoctions for weight loss. Subsequently, some clinical reports and randomized controlled trials investigating these materials have been published.^[9,10]^

Gambisan is an extract of an herbal decoction that was newly developed by Kyung Hee University Korean Medicine Hospital (KHUKMH; Seoul, Korea) in 2009; since then, ≥10 million packs have been prescribed to the target population: patients who are overweight and those with obesity. The recipe was modified from Wolbigachul-tang, which is recorded in the classic traditional Chinese medicine literature, “Keumgueyoryak”; the original prescription was used to treat edema and, more recently, obesity.^[11]^ In 3T3 adipocytes from obesity-induced mice, Gambisan and its 3 major components – *Ephedra intermedia Schrenk*, *Atractylodes lancea (Thumb.) DC*., and *Thea sinensis L.* – suppressed adipogenesis depending on concentration, and the compound demonstrated better results compared with its individual components.^[12]^ In a retrospective chart review involving 28 patients with musculoskeletal pain, Gambisan significantly relieved pain followed by weight reduction.^[11]^ However, there have been no practical studies focusing on its effectiveness for weight loss. Therefore, the present study investigated its weight-reducing effect, as well as reported AEs by reviewing the medical records of adults treated with Gambisan who were overweight, as well as those with obesity.

## 2 Methods

### 2.1 Study design and setting

This study reviewed the medical records of outpatients who were overweight and those with obesity in KHUKMH who were prescribed Gambisan between January 2011 and December 2015. The research plan of this retrospective chart review was approved by institutional review board of KHUKMH (KOMCIRB-160418-HR-016). The data were collected and analyses were conducted until May 2016. One researcher (DHJ) fully reviewed the following sources for data: the Order Communicating System (OCS)—a computerized archive of individual management that records diagnosed disease names, diagnostic tests and therapeutic interventions in a form of a code for billing purposes; and scanned clinical charts of each patient on which the medical information and the progress of the patient were manually recorded, and the printed reports of bioelectrical impedance analysis were present. Given the retrospective nature of this study and the use of anonymized patient data, requirements for informed consent were waived.

### 2.2 Patients

Outpatients who had ever been prescribed Gambisan that is registered in the OCS as code “HH911G”, and met the following criteria were eligible for the present review: the reports of bioelectric impedance analysis (InBody 720, InBody Co., Seoul, Korea) that is registered in the OCS as code “35BL3” were recorded more than 2 times in an interval of 15 to 128 days; age ≥18 years; body mass index (BMI) ≥23 kg/m^2^; and compliance ≥70%.^[5,13]^ In this study, the operational definition of compliance according to the researchers was drawn by dividing the number of days on which Gambisan was prescribed by the total number of days between baseline and endpoint; this was represented as percentage (%).

Patients with any of the following conditions were excluded: taking anti-obesity medication within the previous 6 months; bariatric surgery within the previous 60 months; any medication considered to impact weight (newly applied or changed within the previous 3 months); change in smoking habits within the previous 3 months; psychological eating disorder; breast feeding or the possibility of becoming pregnant; any medical condition (s) affecting weight.

When unit-of-analysis issues arose in this review that assessed bioelectric impedance analysis repeatedly (i.e., 2 or more times during the treatment period), the last measurement satisfying the inclusion criteria was analyzed – and, this was presented as the data of endpoint. Moreover, if a patient attended several sessions, only data from the first session were assessed.

### 2.3 Intervention

Gambisan, a novel granular herbal extract, was officially licensed in Korea in January 2010 (Registration No.: 4008119620000), and quality control of the preparation processes of each of the herbs were conducted according to Korean Good Manufacturing Practice. Comprising this formulation are the herbal part of *Ephedra intermedia Schrenk, Gypsum Fibrosum*, the rhizome part of *Atractylodes lancea DC* and the leaf part of *Thea sinensis L*. Their voucher specimens are stored at KHUKMH. Well-ground ephedra, gypsum and atractylodes are mixed in a ratio of 3:4:2 (w/w) and extracted 2 times by boiling with distilled water. In succession, this extracted liquid undergoes evaporation and lyophilization. Thea separately undergoes an identical process. These 2 powders are mixed in a ratio of 1:0.8 (w/w) into the final product after several homogenizing steps. The standard compound consists mainly of ephedrine, epigallocatechin-3-gallate, and caffeine, with an expected content of 20 mg, 110 mg, and 65 mg, respectively, per 3 g pack. Subjects routinely took a pack of Gambisan 2 to 5 times daily, before each meal. On the first visit, practitioners gave identical information to patients by providing a booklet with brief usual care advice on diet and exercise habits, and with the list of expected AEs including nausea, palpitation, and sleep disturbance, among others in order to prevent patients from feeling anxious and suspicious about intervention due to AEs.

### 2.4 Weight-reducing effect

The primary outcome measure of this study was change in the mean BW of the study population, which was assessed with the reports of the InBody device and analyzed using paired t-tests. Change in the mean percentage body fat (PBF) was also reviewed. In addition, percentage change (%) in BW and percent point change (%p) in PBF of a single patient were operationally defined as the magnitude of effectiveness and were entered into multiple regression models as dependent variables. For practitioners, the adequacy of a specific pharmaceutical intervention is determined by whether it leads to modest weight loss (MWL, i.e., 5% to 10%), as well as by whether it reduces the risks for metabolic and cardiovascular diseases—or improves comorbidities—within 3 months.^[8]^ In this analysis, the proportion of patients who lost more than 5% of BW at baseline was mainly reported in the context of MWL; the proportion of patients who showed ≥ 3% and ≥10% of weight reduction was also assessed.

### 2.5 Subgroup analyses

The following subgroup analyses were conducted according to the conditions of Gambisan prescription:

(1) body mass index^[8]^ (23 kg/m^2^ ≤ BMI < 25 kg/m^2^: overweight, 25 kg/m^2^ ≤ BMI < 30 kg/m^2^: obese I, 30 kg/m^2^ ≤ BMI < 35 kg/m^2^: obese IIa, 35 kg/m^2^ ≤ BMI: obese IIb);
(2) sex;
(3) prescribed duration (4 weeks: 15 to 42 days, 8 weeks: 43 to 70 days, 12 weeks: 71 to 98 days and 16 weeks: 99 to 126 days); and
(4) daily dose (1 time, 2 times, 3 times, 4 times, and 5 times). Paired *t* tests were conducted in each of the subgroups in order to identify any conditions under which Gambisan failed to induce differences in BW (kg) and PBF (%).

### 2.6 Self-reported symptoms

Self-reported symptoms, such as AEs and loss of appetite, were collected by reviewing clinical charts and conducting telephone interviews. Practitioners conducted telephone interviews on a yearly basis with outpatients who completed the full course of intervention in the year, for the purpose of understanding patients’ experience with a main focus on the symptoms associated with Gambisan, experienced at least once by the patients. The patients were asked about loss of appetite and the presence of any unfavorable events and specific symptoms — a list of potential AEs was provided whenever necessary. Measures taken to manage the AEs, their impact, and the results were additionally recorded to grade each AE according to the Common Terminology Criteria for Adverse Events (CTCAE) version 4.03.^[14]^ The frequencies of loss of appetite and AEs were reported and the severities of those AEs were described using grade classification.

### 2.7 Statistical analysis

Statistical analyses were performed using PASW Statistics version 18.0 (SPSS Inc., Chicago, IL). Continuous variables are presented as mean ± standard deviation (SD) and categorical variables are presented as number and percentage. To compare mean BW and PBF at endpoint with baseline, a paired *t* test or a Wilcoxon signed rank test was performed according to whether the data were normally distributed or skewed; *P* ≤ .05 was considered to be statistically significant.

Multiple linear regression analyses were performed to investigate whether any of conditions of Gambisan prescription (BMI, sex, days of intervention and daily dosage) exercised influence on change in BW (%) or PBF (%p). Age was also considered to be a confounding factor. Variables with a variance inflation factor ≥2.5 were regarded to be collinear and were excluded from the regression procedure. Non-significant factors (i.e., *P* > .05) were sequentially eliminated from the model until all existing factors significantly explained the variances of the dependent variables.

## 3 Results

### 3.1 Study population

**Figure 1:**
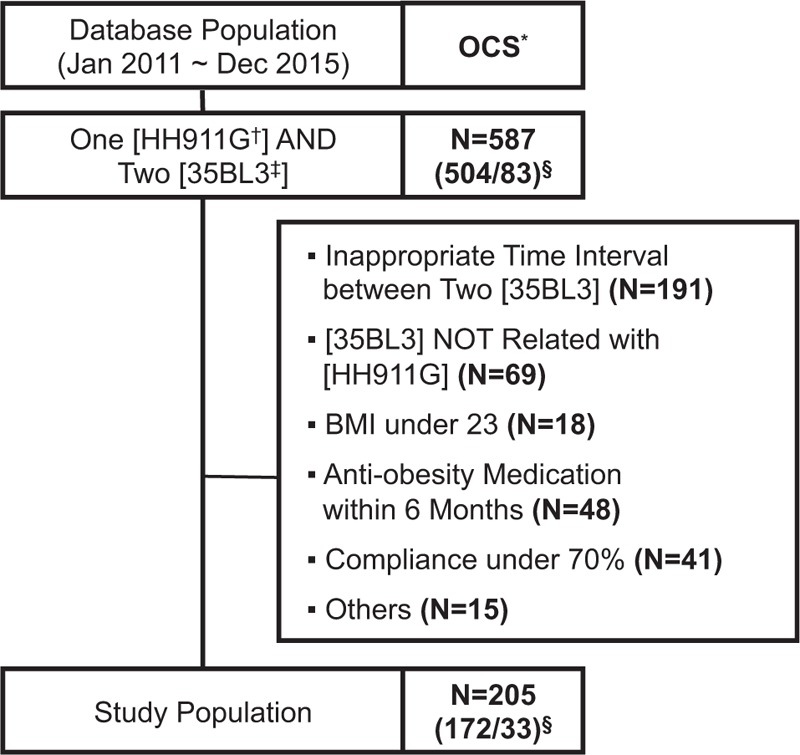
Flow chart of the process to extract the study population from the database of Kyung Hee University Korean Medicine Hospital. ∗ OCS: Order Communicating System (an electronic database of medical records). † HH911G: a code assigned to Gambisan in the OCS. ‡ 35BL3: a code assigned to InBody, a bioelectric impedance analysis, in the OCS. § Number (female/male) of patient identification numbers extracted from the OCS with a search code of ‘One of “HH911G” AND two or more of “35BL3”’.

From the OCS archive, 587 patient IDs that had more than one of the code “HH911G” and more than 2 of the code “35BL3” between January 2011 and December 2015, were extracted. Satisfying the inclusion criteria, 205 patients were finally confirmed as the study population after screening their records (Fig. 1). Among the study population, females were dominant (n = 172 [83.9%]). Mean weight was 73.69 ± 14.49 kg and mean BMI was 28.17 ± 3.67 kg/m^2^. On average, subjects took Gambisan 3.3 times daily (range, 1–5 times daily) for 60.7 days (range, 16–126 days). Detailed demographic and clinical characteristics of the study population are summarized in Table 1.

**Table 1:**
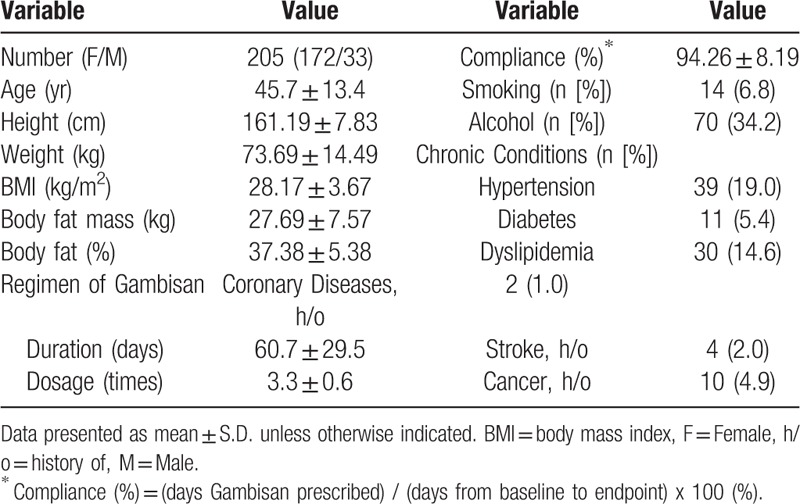
Characteristics of the study population at baseline.

### 3.2 Weight-reducing effect

After the intervention, a significant reduction in mean BW (73.69 ± 14.49 kg to 69.01 ± 13.20 kg [6.15%]; *P* < .001) was observed. Mean PBF was also significantly decreased from 37.38 ± 5.38% to 34.50 ± 5.83% ([2.88%p]; *P* < .001). In the context of MWL, 111 (54.1%) patients showed ≥5% of BW reduction, while 154 (75.1%) lost ≥3%, and 35 (17.1%) lost ≥10% (Table 2).

**Table 2:**
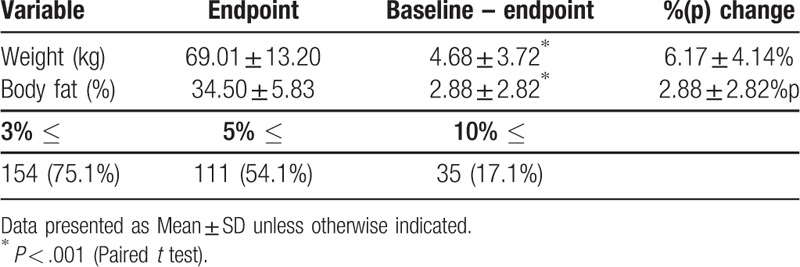
Changes in clinical parameters and the proportion of patients who exhibited ≥3%, ≥5% and ≥10% weight reduction.

### 3.3 Subgroup analyses

**Figure 2:**
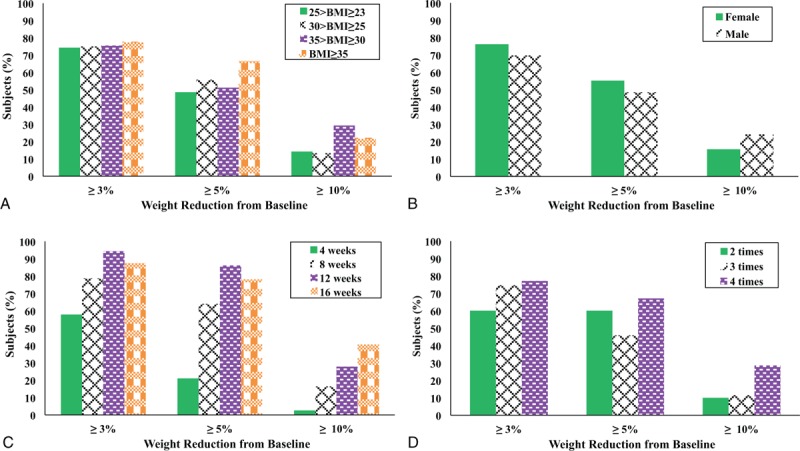
Proportion of patients who showed ≥3%, ≥5%, and ≥10% of weight reduction compared with baseline in subdivisions based on conditions of Gambisan treatment: (A) body mass index (BMI) score; (B) sex; (C) days of Gambisan treatment; (D) daily dose of Gambisan.

Individual patients were assigned to 1 of 4 subgroups based on their weight status. All overweight (*P* < .001; 5.61%), obese I (*P* < .001; 5.83%), obese IIa (*P* < .001; 7.50%) and obese IIb (*P* = .008; 6.41%) groups exhibited significant reduction in mean BW (Table 3). Changes in PBF were also statistically meaningful at endpoint, and the proportion of patients who achieved MWL was 48.6% (overweight), 55.8% (obese I), 51.2% (obese IIa) and 66.7% (obese IIb) (Fig. 2A). Both females and males demonstrated significant decrease in mean BW (female: *P* < .001 [6.42%]; male: *P* < .001 [6.10%]) and PBF (Table 3). Additionally, the success rate of MWL was 55.2% in females and 48.5% in males (Fig. 2B). In subgroup analysis according to the length of treatment, mean BWs were significantly changed in the 4 week group (*P* < .001 [3.72%]), the 8 week group (*P* < .001 [6.67%]), the 12 week group (*P* < .001 [8.36%]) and the 16 week group (*P* < .001 [8.48%]) as well as mean PBFs (Table 3). The success rates of MWL of these subdivisions were 21.1%, 63.9%, 86.1%, and 78.1%, respectively (Fig. 2C). Although the daily consumption of Gambisan ranged from 1 to 5 doses, practitioners rarely recommended patients to take Gambisan 1 time (1 patient) or 5 times (2 patients) per day; thus, these groups were excluded from analysis. In the context of BW reduction, the groups of the 2 time (*P* < .0015 [4.61%]), 3 time (*P* < .001 [5.63%]) and 4 time (*P* < .001 [7.28%]) generated significant changes, and 60%, 45.9%, and 67.1% of subjects in each subdivision exhibited MWL (Fig. 2D).

**Table 3:**
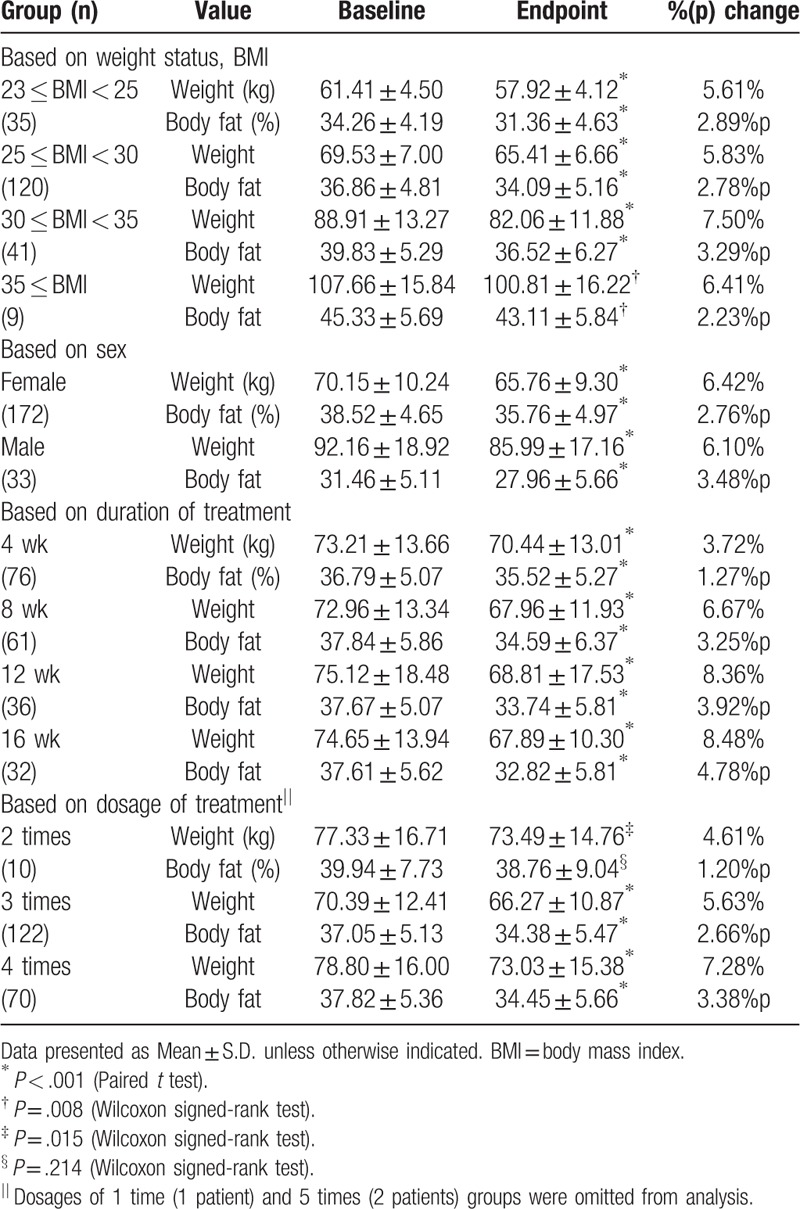
Changes in weight and percentage body fat with 4 subdivision criteria based on clinical considerations for Gambisan prescription.

### 3.4 Multiple linear regression analysis

To assess correlations between the conditions of Gambisan prescription (i.e., BMI, sex, treatment duration and dosage) and the magnitude of BW reduction (%), multiple regression analysis was performed (Table 4). Age was additionally considered to be a confounding factor. Following the elimination procedure, female sex (*P* = .936) and baseline BMI (*P* = .140) were progressively removed because they were not significant contributors to the change in BW. Days of treatment (*β* = .066, *P* < .001), daily dosage (*β* = .908, *P* = .042), and age (*β* = –.087, *P* < .001) demonstrated significance in explaining variances of outcomes and thus were retained in the final regression equation model (R^2^_adjusted_ = .326). An identical procedure was executed to explore the influence of conditions on changes in PBF (Table 4). A backward elimination procedure discarded the variables of baseline BMI (*P* = .611), female sex (*P* = .146) and age (*P* = .129), while days of treatment (*β* = .043, *P* < .001) and daily dosage (*β* = .782, *P* = .013) were retained in the significant final model (R^2^_adjusted_ = .231).

**Table 4:**
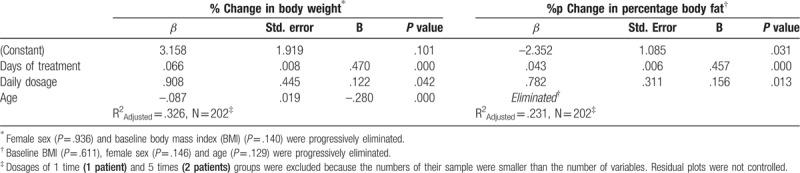
Multiple regression analyses of the impact of conditions of Gambisan prescription and of confounding factor (age) on body weight and percentage body fat reduction by the backward elimination procedure.

### 3.5 Self-reported symptoms

Among the study population, data from 139 patients clearly demonstrated whether the patients experienced loss of appetite or any AEs. Seventy-nine (56.8%) subjects had ever lost appetite during the treatment period. A total of 120 AEs were reported in 69 (49.6%) patients, and nausea (18.7%), palpitation (13.7%), insomnia (10.8%), dry mouth (9.4%), constipation (7.2%), stomach pain (4.3%), and dyspepsia (3.6%) were observed in ≥5 patients (Table 5). Most AEs were classified into mild (grade 1) according to the CTCAE criteria. There were 6 AEs that were regarded as moderate (grade 2); 1 was urinary retention, which led to altering the course of medical treatment of benign prostate hypertrophy, another was dry mouth requiring copious amounts of water; and the other 4 consisted of moderate difficulty falling asleep that did not require taking medication.

**Table 5:**
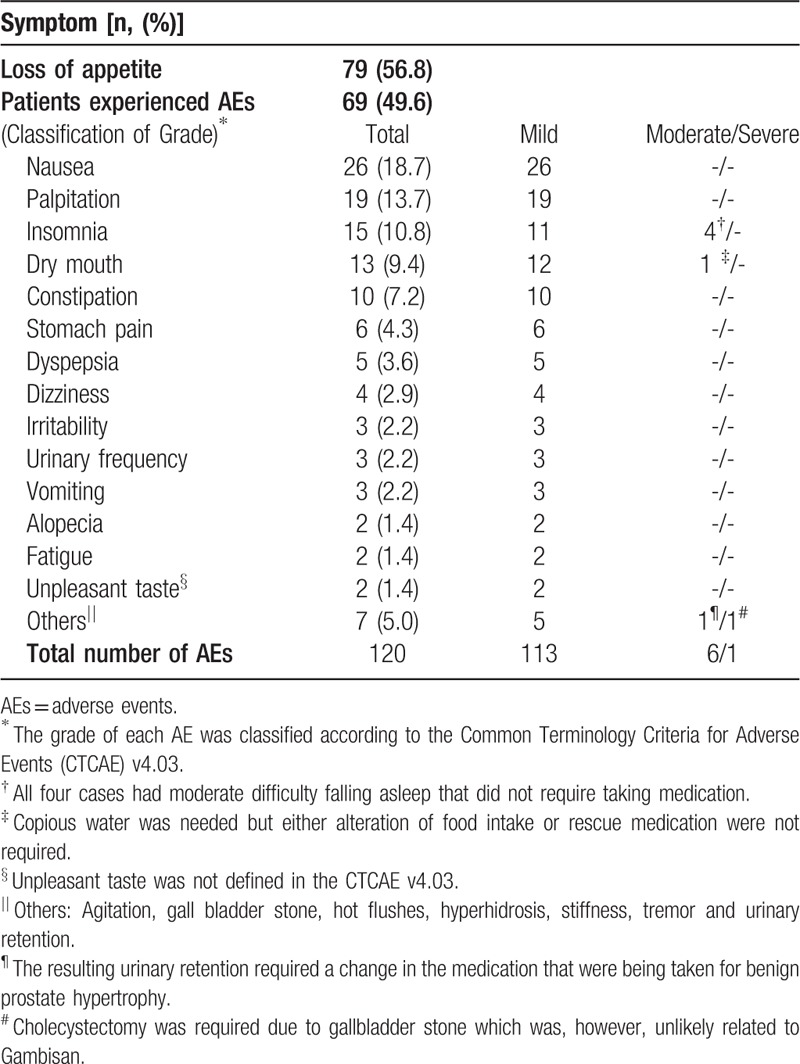
Self-reported symptoms collected from medical chart review and follow-up data from telephone interview of 139 available patients.

## 4 Discussion

Since its development in 2009, millions of packs of Gambisan have been prescribed in KHUKMH for weight loss. By retrospectively reviewing and analyzing the accumulated medical records on an electronic database and scanned clinical charts, the present study tried to explore the effectiveness of Gambisan in weight loss. The results showed that Gambisan induced significant reduction in the mean BW and mean PBF of 205 individuals who were overweight or exhibited obesity. Analysis revealed an average weight loss of 4.68 ± 3.72 kg (6.15%) among the study population following 60.7 days of treatment with Gambisan. This result represents a higher degree of weight loss than previously shown in in 2 retrospective studies (phentermine,^[7]^ orlistat^[15]^) on existing drugs and one non-RCT (Bangpoongtongsungsan^[16]^) and one pilot-RCT (TJ001^[17]^) on herbal medicine, thus may potentially be considered clinically meaningful. In the context of modest weight loss, it is notable that our observed proportion of the study population that lost 5%/10% of BW is comparable to results in the literature, including a meta-analysis, which summarizes the results of RCTs investigating the use of orlistat, sibutramine, or rimonabant over 1 or 2 years (Gambisan: 54.1%/17.1% vs orlistat: 54%/26%; vs sibutramine: 55%/28%; vs rimonabant: 51%/26%).^[18]^

Subgroup analyses were performed to assess the impact of Gambisan on weight loss under various conditions. Practitioners at the KHUKMH recommended monthly follow-ups for 3 to 4 months during this pharmaceutical intervention,^[7]^ and the duration of treatment was categorized into 4 subgroups. The agent induced significant changes in mean BW (kg) and PBF (%) in all pairs classified according to demographic characteristics such as weight status and sex. Moreover, the effect size was not considerably different between subgroups according to change in BW (%), PBF (%p), and the proportion of subjects with MWL, which was though not statistically reviewed. This aspect was identified in the multiple regression procedure that eliminated those 2 factors for being not significant in explaining the dependent variable, all of which indicated this novel herbal medicine could be extensively applied to the overweight population. In terms of treatment, significant reduction in BW (kg) and PBF (%) was observed throughout all pairs based on treatment duration and daily dose of Gambisan. It is noteworthy that the impact was not even, but increased in the magnitude of BW (%) and PBF (%p) change over the subdivisions, which was also not statistically verified. Moreover, this aspect was statistically confirmed by the multiple regression models, which accepted linear correlations of the variables of treatment days and daily dose with the dependent variables. Because the extracted data were not thoroughly controlled in a prospective manner, interpretation of these results is inherently preliminary. However, researchers suggest that practitioners could consider applying this novel herbal medicine for at least 12 weeks and up to 4 times per day, to achieve sufficient effect, provided that any issues concerning AEs are trivial.

For decades, studies to discover the potential of plants and their phytochemicals in controlling obesity have been conducted. These focused on several promising modes of actions^[19,20]^ such as inhibition of lipase activity,^[21]^ appetite suppression/satiety induction,^[22]^ thermogenesis, regulation of lipid metabolism, and inhibition of adipogenesis.^[23]^ The mechanisms of Gambisan's anti-obesity effect may be explained by its standardized herbal composition, which includes ephedrine, epigallocatechin-3-gallate, and caffeine. Over the past 20 years, research investigating the mixture of ephedrine/caffeine for the purpose of weight loss has been conducted. This mixture is believed to reverse obesity by reducing food intake^[24,25]^ and increasing energy expenditure^[24,26]^ through thermogenesis,^[27,28]^ mainly in brown adipose tissue via the sympathetic nervous system.^[29]^ Accordingly, self-reported symptoms in this study revealed that 79 of 139 patients (56.8%) experienced loss of appetite. Epigallocatechin-3-gallate, a major polyphenol present in green tea, has been proven to have a regulatory effect on fat accumulation by inhibiting the activity of pancreatic lipase and by reducing fatty acid synthase and acetyl CoA carboxylase-1 mRNA expression.^[30]^ Moreover, it was reported that Gambisan suppresses adipogenesis in 3T3-L1 adipocytes in rats by downregulating the expression of related genes in a synergistic manner.^[12]^

The occurrence of AEs was common in this study; however, they were comparable to those of other anti-obesity drugs (Gambisan: 49.6% vs orlistat^[5,31,32]^: 78% to 91%, sibutramine^[33–35]^: 49% to 79%, phentermine^[7,36,37]^: 30% to 96%). In terms of severity of AEs, 6 out of 120 AEs that were associated with Gambisan use were classified as moderate, which meant that the patients experienced some degree of discomfort and in some cases, needed to change their medication for their existed comorbidity. All of these symptoms disappeared within a few days to weeks. Presented in Table 5 without being mentioned in the result part is one case of a patient who underwent cholecystectomy (grade 3 [severe]) due to gallstone development. Considering that the first appearance of related symptoms was about three to four months after the last visit and that gallstone development has not been reported in previous researches about herbal mixtures with ephedrine/caffeine^[38–41]^, researchers think this event was unlikely to be associated with the use of Gambisan. Additionally, there were 2 cases of transient hair loss (Grade 1: mild); however, both cases were followed by extreme voluntary reduction of caloric intake for several weeks regardless of the practitioners’ recommendation. It is difficult to draw robust conclusions regarding the adverse effects of Gambisan from this study, as subjects included in final analyses tend to exhibit higher compliance, and the results of liver and kidney function tests and vital signs were not included. Nevertheless, our results shown in Table 5 indicate that the side effects of Gambisan may be tolerable when administered correctly under the discretion of a medical professional, warranting further investigation into its clinical safety.

The most frequent AEs included nausea, palpitation, insomnia, dry mouth, and constipation, and these AEs were consistent with those reported by previous randomized controlled trials on herbal mixtures with ephedrine/caffeine.^[38–41]^ Over the past few decades, controversies regarding dietary supplements containing ephedrine have emerged.^[42,43]^ However, a series of investigations^[38–41]^ have reported that low-dose ephedrine, alternatively enhanced with caffeine, did not significantly induce more unfavorable effects compared with placebo control. Furthermore, the study population in the present study took the same—or less—of these components than previous researches as Gambisan includes 20 mg of ephedrine and 65 mg of caffeine in each 3 g pack. Additionally, as a formulation of Traditional Korean Medicine, which has specific combination principles that account for the complexity of interacting systems of many networks in the human body,^[44]^ gypsum, which is expected to alleviate hyperactivate nervous symptoms and atractylodes, which alleviates digestive symptoms, were added.

Aside from the occurrences of AEs, there were unavoidable limitations to this study given that it was a retrospective chart review. First, the study population was restricted to patients who were consistently prescribed Gambisan and who underwent bioelectric impedance analysis measurement more than 2 times. Thus, those with low compliance due to unsatisfactory improvement or AEs were more likely to be excluded from the review, which likely introduced selection bias in this study. Second, in the outpatient clinic, patients who visit KHUKMH with the purpose of weight reduction have been routinely prescribed Gambisan, or an herbal medicine of similar composition. Thus, it was impossible to generate a comparative control group for the study from available databases, such that the results of this preliminary study are not confirmatory. Third, limitations in the electronic medical charts provided incomplete data regarding patients’ economic status and dietary and exercise habits, all of which are important obesity-related factors. Finally, to determine the detailed properties of Gambisan, laboratory and anthropometric parameters related to energy and fat metabolism must be investigated. In the context of the above limitations, a double-blinded, randomized, placebo-controlled, phase 2 study is planned based on the findings of this study; this has been registered^[45]^ by the authors of the present study.

In conclusion, the present study found that Gambisan was effective for weight loss regardless of prescription conditions and its safety was competitive in terms of AEs. However, the results should be interpreted with caution because the study design was not confirmative and had several limitations. Further well-designed, prospective studies are required to confirm the findings of this study with regard to the promising novel anti-obesity herbal medicine.

## Acknowledgments

The authors are indebted to Dr. Hyunho Kim for his helpful comments on statistical analyses.

## Author contributions

This study project was administrated and supervised by the corresponding author, Jae-Dong Lee. Seunghoon Lee and Dae-Hyun Jo conceived and designed the study. DHJ transformed the data from medical records and conducted the statistical analyses. DHJ drafted the report and SL wrote the final manuscript. Thus, DHJ and SL contributed equally. All authors participated in the discussion and the critical revisions of the initial article and approved to submit the final manuscript.

**Conceptualization:** Dae-Hyun Jo, Seunghoon Lee.

**Formal analysis:** Dae-Hyun Jo.

**Methodology:** Dae-Hyun Jo, Seunghoon Lee.

**Project administration:** Jae-Dong Lee.

**Supervision:** Jae-Dong Lee.

**Writing – original draft:** Dae-Hyun Jo.

**Writing – review & editing:** Dae-Hyun Jo, Seunghoon Lee, Jae-Dong Lee.
